# The use of brain tissue mechanics for time since death estimations

**DOI:** 10.1007/s00414-023-03068-0

**Published:** 2023-08-16

**Authors:** Johann Zwirner, Pavithran Devananthan, Paul Docherty, Benjamin Ondruschka, Natalia Kabaliuk

**Affiliations:** 1 Institute of Legal Medicine, University Medical Center Hamburg-Eppendorf, Hamburg, Germany; 2 Department of Oral Sciences, University of Otago, Dunedin, New Zealand; 3 Department of Mechanical Engineering, University of Canterbury, Christchurch, New Zealand; 4 Bimolecular Interaction Centre, Christchurch, New Zealand

**Keywords:** Biomechanics, Brain, Rheometry, Post mortem interval, Time since death estimation

## Abstract

Time since death estimation is a vital part of forensic pathology. Despite the known tissue degradation after death, the efficacy of using biomechanical tissue properties to estimate time since death remains unexplored. Here, eight brain tissue localizations were sampled from the frontal lobe, parietal lobe, anterior and posterior deep brain, superior colliculi, pons, medulla, and cerebellum of 30 sheep; were then stored at 20 °C; and subsequently subjected to rheometry tests on days zero to four after death. Overall, the measured tissue storage modulus, loss modulus, and complex shear modulus decreased after death for all of the tested regions in a site-specific manner. Day zero to day one changes were the only 24-h interval, for which statistically significant differences in tissue mechanical moduli were observed for some of the tested brain regions. Based on receiver operator characteristic analyses between day zero and the pooled data of days one to four, a post mortem interval of at least 1 day can be determined with a sensitivity of 90%, a specificity of 92%, and a positive likelihood ratio of 10.8 using a complex shear modulus cut-off value of 1461 Pa for cerebellar samples. In summary, biomechanical properties of brain tissue can discriminate between fresh and at least 1-day-old samples stored at 20 °C with high diagnostic accuracy. This supports the possible value of biomechanical analyses for forensic time since death estimations. A striking advantage over established methods to estimate the time since death is its usability in cases of disintegrated bodies, e.g. when just the head is found.

**Supplementary Information:**

The online version contains supplementary material available at 10.1007/s00414-023-03068-0.

## Introduction

Time since death estimation is a vital part of forensic practice. Routine methods include measurements and analyses of the rectal temperature [1, 2], lividity [3], rigor mortis [4], and the electrical excitability of the facial muscles [5]. There are numerous confounding factors on all available methods, which lead to reporting a suspected time since death interval rather than a determined point in time [6]. There are only a few specific cases wherein the time since death is determined exactly in the absence of witnesses, e.g. when evaluating the readings of cardiac implantable electronic devices [7]. Utilising changes in biomechanical properties of certain tissues after death is yet to be explored for forensic time since death estimations. For biomechanical analyses aiming to estimate the time since death, it is crucial to choose appropriate tissue types for post mortem time frames of interest. From anthropological and autopsy experiences, skeletal remains might stay somewhat intact up to one century [8] whereas many soft tissues decompose in a matter of hours to days [9]. From the authors’ experience, brain tissue degrades in a matter of hours to a few days at ambient temperatures, making it difficult to prepare slices for routine macroscopic and microscopic analyses even at comparatively short post mortem intervals (PMIs). Previous post mortem studies on brain tissue of different species [10–14] including human beings [15–18] predominantly focused on obtaining the mechanical properties as close to the in vivo conditions as possible and, therefore, kept the PMIs short and/or the samples cool until testing. 

For forensic time since death estimations, it is critical to biomechanically investigate brain tissue after being stored at different ambient temperatures including the common ambient temperature of approximately 20 °C. From casework experience, ambient temperatures of approximately 20 °C are very common for cadavers found indoors, for which time since death analyses are of high forensic interest.

This research provides the first strategical analyses of the correlation between several biomechanical properties of brain tissue and the time since death. It was aimed at probing the concept that biomechanical properties can provide valuable information for time since death estimations. For this, ovine abattoir tissues were used for which the time of death was known and the subsequent ambient conditions were strictly controlled.

## Materials/methods

### Tissue collection and preparation

**Fig. 1:**
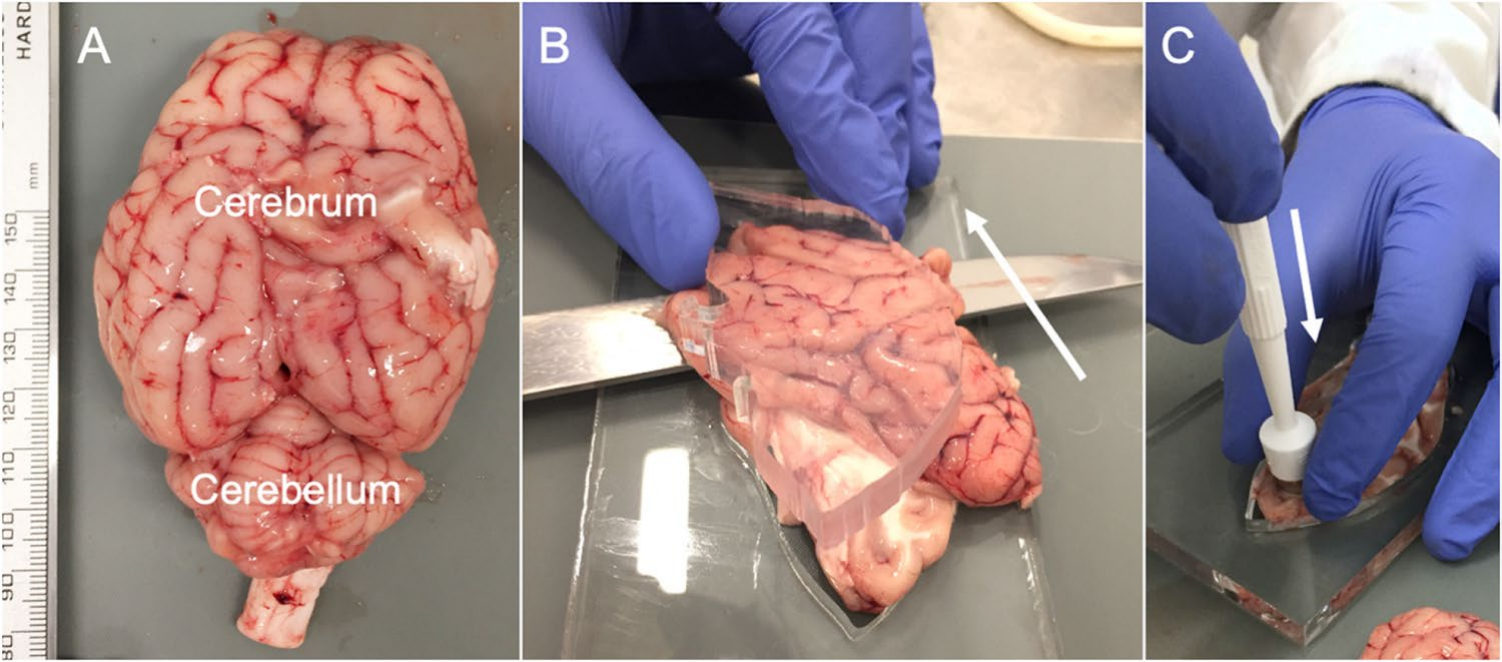
**A** The ovine brain is shown after retrieval. **B** The brains were cut along the sagittal axis (arrow) using a customised 8-mm-thick laser-cut acrylic stencil. **C** Cylinders were punched from the different brain regions shown in Fig. 2 using a biopsy punch

**Fig. 2:**
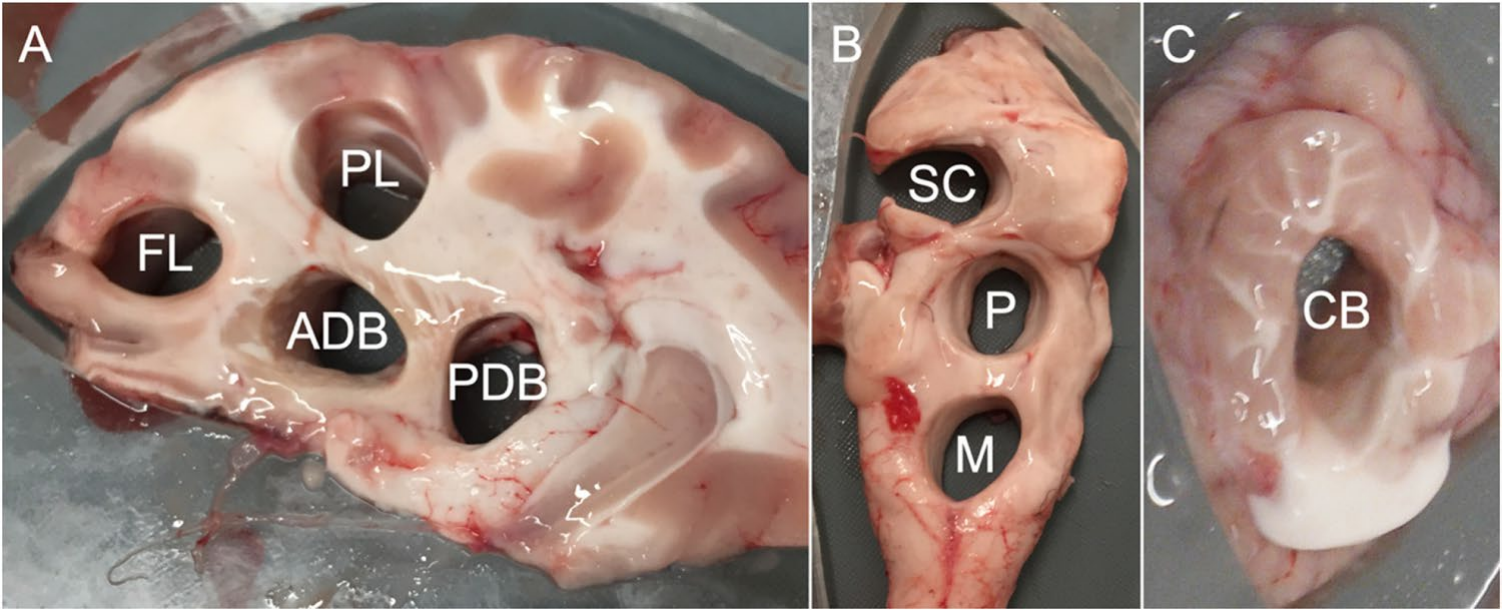
The punching sites of the different samples are shown for the cerebrum (**A**) and midbrain and brainstem (**B**) as well as the cerebellum (**C**): ADB anterior deep brain, CB cerebellum, FL frontal lobe, M medulla, P pons, PDB posterior deep brain, PL parietal lobe, SC superior colliculi

**Fig. 3:**
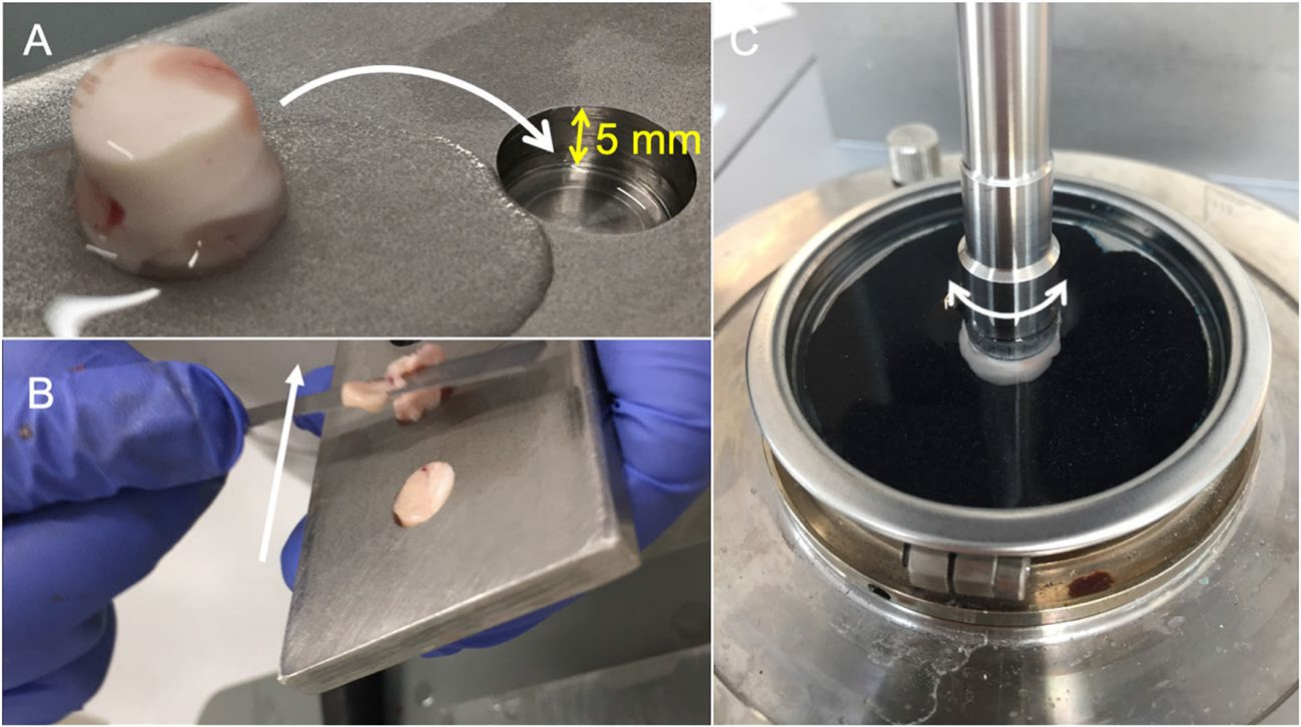
**A** The initially punched sample cylinder was transferred to a cutting mould to adjust its height to 5 mm. **B** For this, a sharp microtome blade was used to cut horizontally (arrow). **C** The samples were tested submerged in saline solution at 20 °C

A total of 30 abattoir ovine full brains including cerebral and cerebellar hemispheres, midbrains, pontes, and medulla were procured “still warm” within 2 h of sacrifice. The animals were sacrificed for meat, for which the collected tissues were a waste product. Hence, obtaining ethical approval for this study was not applicable. The animals were sacrificed by throat slitting, thereby not damaging the brain tissue. Immediately following retrieval from the skull, the brains were rinsed in normal saline to remove all blood components and debris resulting from the retrieval process. Thereafter, the brains were placed in containers and fully submerged in normal saline at 20 °C until mechanical testing to keep the tissues moist. Testing on day 0 was finished within 4 h after the animals were sacrificed. Five groups were created according to the five testing points in 24-h intervals between days 0 and 4. Per testing day, the maximum sample size (*n*) was 12 for paired (e.g. hemispheres) and 6 for unpaired (e.g. medulla) brain sites (e.g. 60 cerebral hemispheres in total for 5 different testing groups resulting in 12 hemispheres for each individual testing day). An equal ratio of right and left samples was assured for each paired brain site. Right before mechanical testing, eight types of testing samples were prepared in a standardized manner according to their location to ensure the highest possible degree of similarity. In particular, care was taken to ensure the proportion of grey and white matter was as consistent as possible within sample locations. The cerebral hemispheres were cut along the sagittal axis using a microtome blade and a customised 8-mm-thick laser-cut acrylic stencil. Thereby, a medial brain slice was created, on which the deep brain structures such as the basal ganglia could be defined. From this slice, a frontal lobe (FL) sample and a parietal lobe (PL) sample as well as an anterior (ADB) and a posterior deep brain (PDB) sample were punched using a disposable biopsy punch with a diameter and height of 10 mm, respectively (Fig. 1). For the cerebellar samples (CB), the stencil was used in the same way as for the cerebrum to create lateral slices, which were then punched according to the cerebral samples. From the midbrains, the left superior colliculi (SC) were punched in a posterior-anterior direction. Respectively, pons (P) and medulla (M) samples were hollow punched. An overview of all sample sites is given in Fig. 2. Following punching, all samples were trimmed to a height of 5 mm using a microtome blade and a customised mould (Fig. 3). For trimming, the flat sample surfaces created during slicing with the stencil were placed face down into the mould to ensure the resulting cylindrical sample had flat faces.

### Biomechanical testing

Testing was performed using a rheometer (MCR302; Anton Paar, Graz, Austria). When the tissue was placed into the apparatus, it was placed under an axial preload of 0.1 N to ensure adhesion to the contact surfaces, fully submerged in saline, and given 100 s to relax. To reduce sample slippage during testing, sandpaper was placed between the samples and the rheometer base plate while the contact surface of the rotating measuring tool was sandblasted.

A total of 50 cycles at a peak shear strain of 0.03 (rad) at 3 Hz with a continued 0.1 N compression force were applied to each sample. The 0.03 (rad) strain was found to be a good magnitude to prevent slippage of the tissue during testing. The 3-Hz strain frequency was empirically shown from our preliminary experiments (in data not shown) as the point where the strain frequency dependence was no longer an issue. The tests were performed with samples submerged in normal saline solution maintained at 20 °C.

### Data analysis

The tissue storage, loss, and complex shear moduli were calculated. Microsoft Excel Version 16.65 (Microsoft Corporation, Redmond, USA) and GraphPad Prism version 9 (GraphPad Software, La Jolla, USA) were used for statistical analyses and visualization of the data. The assessment for Gaussian distribution was done using the Shapiro–Wilk normality test. Ordinary one-way ANOVA tests including Tukey’s multiple comparison and Kruskal–Wallis tests including Dunn’s multiple comparison were applied for normally and non-normally distributed data, respectively. *P*-values ≤ 0.05 were considered statistically significant. Side comparisons between the hemispheres as well as a comparison between the pons and the medulla were performed to investigate whether the respective counterparts could be pooled for further analyses. Further comparisons were made between the day 0 values of all regions as well as between the different testing days of each brain region separately for the different biomechanical properties to differentiate very fresh from ‘at least one day death’ samples. Statistical comparisons were only performed if at least six samples were present per group. Lastly, receiver operator characteristic (ROC) curves were created based on the results of the statistical comparisons described above.

## Results

### Overview of the results

**Fig. 4:**
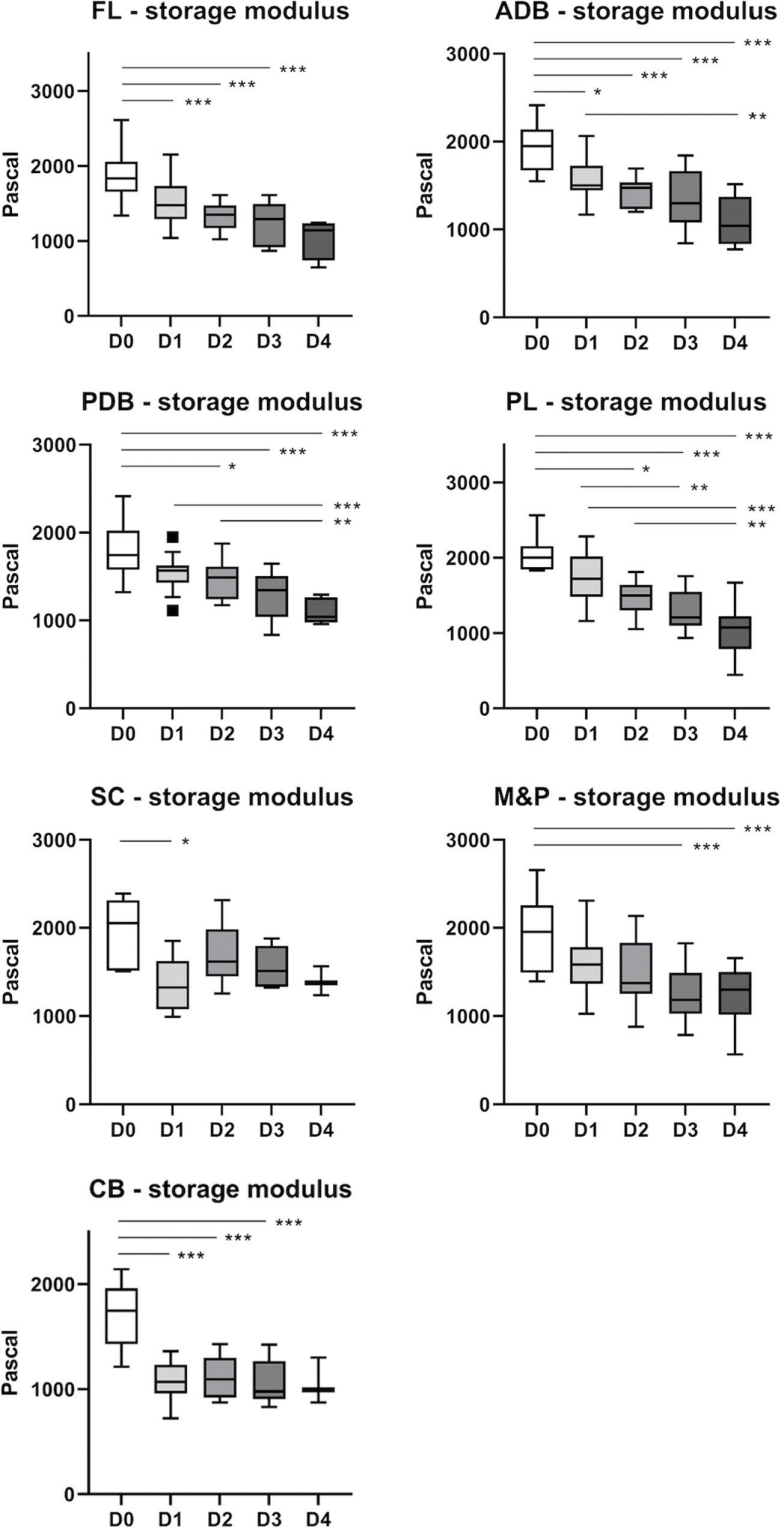
The storage moduli are depicted for the different brain regions over the complete testing time frame of four days: D1–4, days 0 to 4, ADB anterior deep brain, CB cerebellum, FL frontal lobe, M&P pooled medulla and pons, PDB posterior deep brain, PL parietal lobe, SC superior colliculi

No statistically significant differences were observed in biomechanical properties across the left and right hemispheres as well as the values of the pons and the medulla (*p* ≥ 0.071). Hence, data was pooled across the hemispheres as well as the pons and medulla data (M&P) for further comparisons. All pooled samples except for the loss modulus of the PDB, M&P, and CB passed the Shapiro–Wilk normality test. In general, the investigated biomechanical properties of brain tissue decreased between days 0 and 4 for all investigated brain regions in a site-specific manner (Fig. 4).

Several brain regions revealed statistically significant differences in the mechanical moduli between days 0 and 1. The biomechanical properties investigated across the different brain regions were unable to exhibit statistically significant discrimination between days 1 and 2, 2 and 3, or 3 and 4 to a statistically significant threshold.

For 2-day intervals, all but CB and SC showed significant differences of at least one biomechanical parameter when comparing days 0 and 2. The comparison between days 1 and 3 showed significant differences in the storage modulus (*p* = 0.002) and the complex shear modulus (*p* = 0.003) of the PL. The storage modulus and complex shear modulus of days 2 and 4 also differed significantly for both the PDB (*p* ≤ 0.044) and the PL (*p* ≤ 0.007).

For the 3-day interval, all properties differed significantly between days 0 and 3 (*p* ≤ 0.050). The day 1 and 4 comparison revealed significantly different results for the storage modulus (*p* ≤ 0.010) and complex shear modulus (*p* ≤ 0.011) of the ADB, PDB, and PL as well as the loss modulus of the ADB (*p* = 0.048) and the PL (*p* = 0.022).

For the 4-day interval, all properties except the loss modulus of the M&P differed significantly between days 0 and 4 (*p* ≤ 0.005). Tissue degradation made it impossible to punch useable samples for biomechanical testing in several day 3 and 4 cases, for which statistical comparisons had to be omitted. Data for the storage modulus is presented below in more detail. Information on the applied statistical test for the group comparisons as well as the *p*-values for all tested parameters is available in the Supplementary Table 1.

### Storage modulus

The storage modulus showed a steady decrease for the FL, ADB, PDB, and PL from day 0 towards day 4. For the CB, the storage modulus dropped between days 0 and 1 and plateaued until day 4. For the M&P, after showing a steady decrease between days 0 and 3, the median and average value of the storage modulus re-increased between days 3 and 4 on a statistically non-significant level. For the SC, the storage modulus significantly decreased (*p* = 0.024) between days 0 and 1 but then insignificantly increased again between days 1 and 2 with no statistically significant difference between days 0 and 2.

For the FL, ADB, and CB, the storage values of day 0 were significantly different from all other testing days, for which statistical comparisons could be performed based on the number of samples (up to 3 days for the FL and CB and up to 4 days for ADB). A graphical depiction of the storage modulus data of the different brain regions is given in Fig. 4.

### Diagnostic ability of a mechanical property-based discrimination between day 0 and the pooled data of days 1 to 3 or 4

**Fig. 5:**
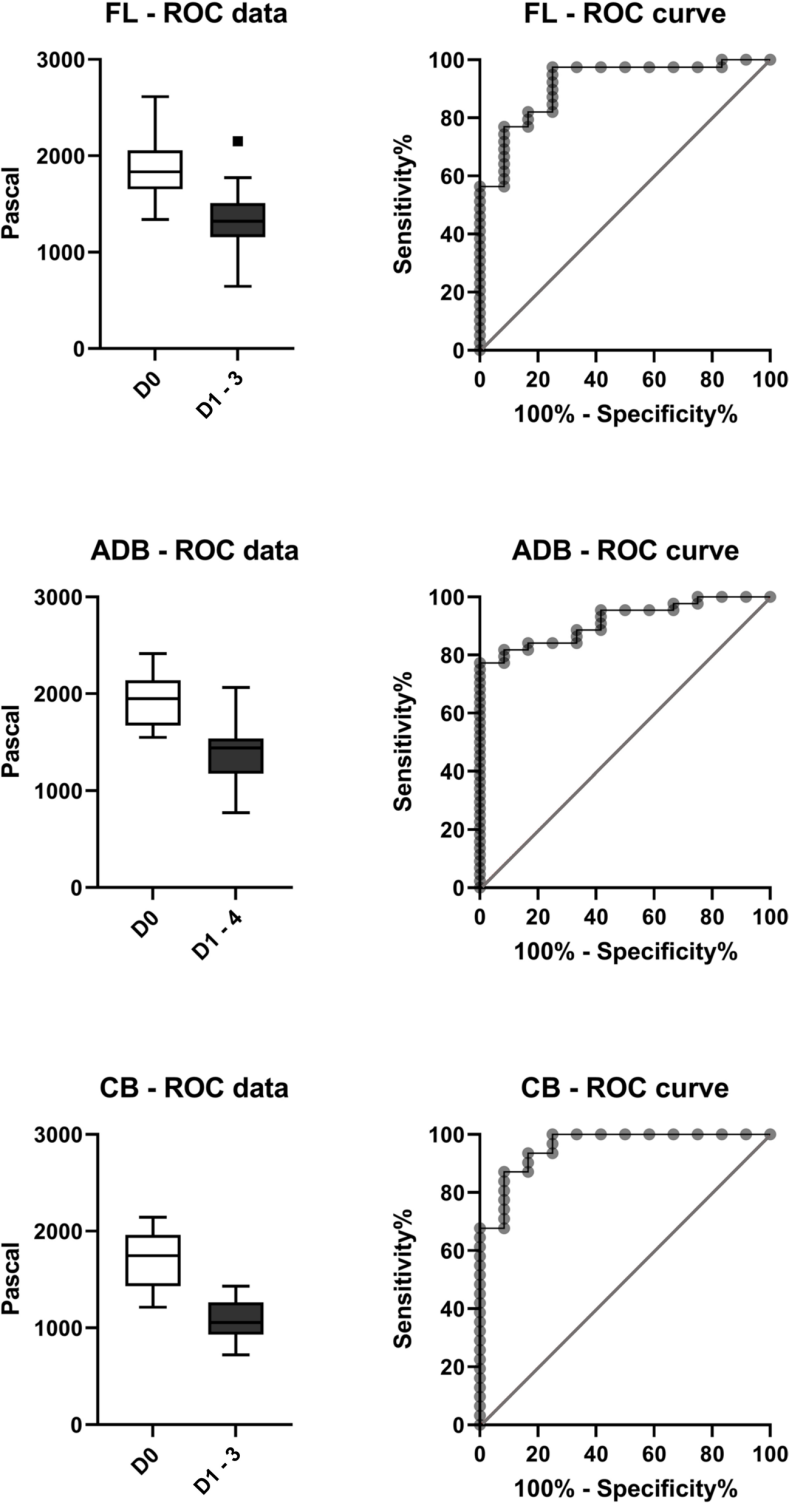
Boxplots of storage moduli and corresponding receiver operator characteristic (ROC) curves of the frontal lobe (FL), the anterior deep brain (ADB), and the cerebellum (CB) across day 0 to pooled data from days 1 to 3/4 (note: no day 4 values were available for the frontal lobe and the cerebellum)

The statistically significant difference between the day 0 values and the other testing days for the FL, ADB, and the CB was observed for both the loss modulus and the complex shear modulus. To adapt this outcome and provide the highest possible discrimination across day 0 values and pooled day 1 to 3/4 values (insufficient number of day 4 values in some cases), ROC curves were plotted. Ideal thresholds were declared by minimising the Euclidian distance to full sensitivity and sensitivity. The ROC results are depicted in Table 1 and Fig. 5.

**Table 1:** The results of the receiver operator characteristic analyses: *CI* confidence interval, *ADB* anterior deep brain, *CB* cerebellum, *FL* frontal lobe. *The 95% CI for the specificity was 64.6–99.6% in all cases

Biomechanical property	Region	Cut-off value	Sensitivity (%)	95% CI (%)	Specificity (%)*	Likelihood ratio
Storage modulus (Pa)	FL	< 1511	76.9	61.7–87.4	91.7	9.2
	ADB	< 1627	81.8	68.0–90.5	91.7	9.8
	CB	< 1320	87.1	71.2–94.9	91.7	10.4
Loss modulus (Pa)	FL	< 551.7	74.4	58.9–85.4	91.7	8.9
	ADB	< 597.4	81.4	67.4–90.3	91.7	9.8
	CB	< 534.6	83.9	67.4–92.9	91.7	10.1
Complex shear modulus (Pa)	FL	< 1607	74.4	58.9–85.4	91.7	8.9
	ADB	< 1758	84.1	70.6–92.1	91.7	10.1
	CB	< 1461	90.3	75.1–96.7	91.7	10.8

### Early post mortem biomechanical properties of ovine brain hemispheres, medulla oblongata, and cerebellum

The day 0 storage moduli, loss moduli, and complex shear moduli of all tested regions are depicted in Tables 2, 3, and 4. A comparison of the day 0 values of all aforementioned corresponding biomechanical properties between the regions only revealed a significantly higher loss modulus of the PL of 785 ± 115 Pa (median = 783 Pa) compared to the CB of 616 ± 80 Pa (median = 599 Pa).

**Table 2:** The day 0 storage moduli of the different brain regions: *ADB* anterior deep brain, *CB* cerebellum, *FL* frontal lobe, *M&P* pooled medulla and pons, *PDB* posterior deep brain, *PL* parietal lobe, *SC* superior colliculi

Storage modulus	FL	ADB	PDB	PL	CB	M&P	SC
Average (Pa)	1905	1927	1795	2033	1706	1924	1968
Median (Pa)	1834	1948	1743	2002	1746	1953	2053
STDEV (Pa)	360	293	307	220	286	413	389
Minimum (Pa)	1338	1550	1322	1829	1215	1395	1507
Maximum (Pa)	2613	2414	2414	2561	2143	2657	2389
*n*	12	12	12	12	12	12	6

**Table 3:** The day 0 loss moduli of the different brain regions: *ADB* anterior deep brain, *CB* cerebellum, *FL* frontal lobe, *M&P* pooled medulla and pons, *PDB* posterior deep brain, *PL* parietal lobe, *SC* superior colliculi

Loss modulus	FL	ADB	PDB	PL	CB	M&P	SC
Average (Pa)	680	693	710	785	616	692	742
Median (Pa)	567	679	705	783	599	639	711
STDEV (Pa)	137	99	101	115	80	161	203
Minimum (Pa)	456	514	557	638	514	474	483
Maximum (Pa)	911	874	900	982	808	1046	1000
*n*	12	12	12	12	12	12	6

**Table 4:** The day 0 complex shear moduli of the different brain regions: *ADB* anterior deep brain, *CB* cerebellum, *FL* frontal lobe, *M&P* pooled medulla and pons, *PDB* posterior deep brain, *PL* parietal lobe, *SC* superior colliculi

Complex shear modulus	FL	ADB	PDB	PL	CB	M&P	SC
Average (Pa)	2023	2048	1931	2181	1816	2046	2105
Median (Pa)	1949	2072	1886	2126	1854	2071	2211
STDEV (Pa)	383	306	317	236	285	436	430
Minimum (Pa)	1414	1633	1469	1940	1334	1510	1583
Maximum (Pa)	2757	2567	2576	2731	2230	2856	2590
*n*	12	12	12	12	12	12	6

## Discussion

### High diagnostic accuracy for discrimination between fresh and at least 1-day-old brain samples

To be beneficial for time since death estimations, the biomechanical properties should discriminate different points in time after death on a statistically significant level. Ideally, particular cut-off values would link the biomechanical properties to indicative points in time after death. The rheometry tests in this study proved to be a suitable method to biomechanically measure brain tissue degradation after death. Day 0 values of the storage modulus, loss modulus, and complex shear modulus revealed statistically significant decreases for all measured points in time after death (Fig. 4). On that basis, ROC analyses were performed to analyse the potential of the applied test to discriminate between day 0 values and the pooled data from days 1 to 3/ 4 (Table 1). Thereby, cut-off values for the FL and ADB as well as the CB were identified, which showed positive likelihood ratios between 9 and 11 for the detection of brain samples with a PMI of at least 1 day. For example, the CB showed positive likelihood ratios between 10.1 and 10.8 for the storage modulus, loss modulus, and complex shear modulus (Table 1).

While based on the here presented results, the binary classification of ‘day 0 or older’ seems very promising, the diagnostic value of brain samples using this approach has poor resolution beyond day one. To achieve sufficient precision in the time since death estimation after day 1, the inter-subject variance in brain tissue properties would need to be reduced. Within the first 24 h, shorter time intervals might provide statistically significant differences applying the here used method and protocol. From a practical standpoint, a significant difference between days 0 and 2 will not necessarily yield specific and valuable insight. In particular, since it is impossible to classify samples from days 1 vs 2 nor 2 vs 3, the ability to determine day 0 to day 2 is redundant. For example, if the ground truth for a sample sat on the 50th percentile for 2 days since death, one would not be able to determine if the specific sample was day 1, day 2, or day 3 with confidence. But it would be possible to determine it was not likely to be day 0. Hence, using brain tissue, the given method lacks the resolution for highly specific prediction of time since death after day 1. However, there may be potential for the findings of this study to be linked to other indicators to provide greater resolution than possible than any single indicator.

### Similar trends between regions but retrieval sites are a major factor

The day 0 value comparison between the regions showed no significant differences in the biomechanical properties within the cerebral samples. However, there were some isolated differences that yielded statistical significance between the cerebral samples and the joint M&P samples as well as the CB. Hence, anatomical locations should be respected for time of death estimation and brain regions should not simply be pooled. The post mortem biomechanical properties over 4 days revealed differences between the various brain regions. For example, the mean values of the storage moduli of the cerebral samples gradually decreased over the 4 days of testing. This is well justified by an ongoing degradation with the tissue’s decreasing potential to store elastic energy. However, for the M&P, as a part of the brainstem, the storage moduli increased between days 3 and 4. In contrast, the SC exhibited an unexpectedly steep and statistically significant drop between days 1 and 2, which then re-increased the subsequent day. The authors hypothesise that the small late increase for the M&P might be caused by small deviations in punching sites due to the sample’s ongoing degradation and the increased difficulty to meet the same spot each time. While the early drop of the storage moduli of the SC seems counterintuitive, it stood up to statistical rigor and yielded a strongly predictive change. Interestingly, the CB storage modulus was the only investigated tissue property that seemed to reach a plateau for values from day 1 onwards following a significant drop from day 0 to 1. All other brain regions seemed to gradually decrease over the four testing days.

### Where the method fits into daily forensic practise

This study showed that the here tested biomechanical properties of brain tissue depend on the PMI and are, thus, potentially valuable for time since death estimations. The authors hypothesise that the given method is temperature-sensitive and would need to be recalibrated and validated for the purpose of assessing PMI of subjects that have been cooled before, e.g. in the morgue. Furthermore, analysing brain tissues is ultimately an invasive process and commonly requires the opening of the neurocranium, which is only done during autopsies. Retrieving brain samples before autopsy poses several ethical and legal challenges. However, time since death estimations are commonly critical in non-natural or unclear deaths such as homicides or suicides, which often require an immediate autopsy. In those cases, the brain tissue is instantly available and can be biomechanically investigated with little delay after arrival in the morgue and potentially even parallel to autopsy. A profound advantage of this given method over traditional methods such as lividity or rigor mortis is its usability in cases of disintegrated bodies, e.g. when just the head is found.

### Implications

This is the first study to reveal the potential of brain tissue mechanics to inform forensic time since death estimations. To make this method suitable for everyday forensic use, several points have to be clarified.

Firstly, it has to be investigated whether human tissues show comparable results to the ovine tissue and whether the observed trends can hold. The authors assume that ovine and human brain tissues will perform similarly under the testing method described above. Human and ovine brains have a similar structural composition and a proximal size. However, in-depth validation is an important next step.

Secondly, it is crucial to determine to what extent temperature alters the respective biomechanical properties. We know that cooling reduces the decomposition of biological tissue, and brain tissue in particular [19]. Therefore, it may be expected that the conclusions derived from the biomechanical properties described in this study must be adjusted for temperature.

Thirdly, the results of this study show that brain tissue is of use for time since death estimations after 24 hrs. Given the gradual decrease of most biomechanical properties of some brain regions, it might even be possible to declare that exceptionally low values are unlikely reached before a particular PMI has passed. For example, several low storage modulus values of the PL were only present in day 4 samples. Therefore, there is a potential use for brain biomechanics beyond day 1 estimations.

Finally, it may be possible that anthropometric data from the deceased could be used to augment the interpretation of the rheometric properties and improve time since death estimations.

### Limitations

This study was limited in sample population. Nonetheless, many comparisons exhibited statistical significance. Increasing the sample size is likely to achieve increased rates of statistical significance. However, the ROC values are the most important tool for assessing classification potential and are unlikely to improve with increased population size. The ROC values are likely to improve if the sample location was more consistent, which is challenging from a practical perspective. Attempts were made to meet the same anatomical locations for sample harvesting each time. Due to different brain sizes and slight anatomical deviations, the underlying rheological properties may have contributed to the high standard deviations observed here for some sampling sites. Even though attempts were made to keep the temperatures constant throughout the analysis, slight temperature deviations between the tested samples might have influenced the results. It is assumed that the brains investigated in this study were retrieved from healthy sheep. The brains were not tested for medical conditions, which might have decreased the precision of the biomechanical properties investigated. However, pathological or traumatic changes were not visual on gross inspection. Tissues were kept in normal saline solution for storage and testing, which might have influenced the stated results. Even though attempts were made to respect strict 24-h time intervals between the different testing days, slight deviations (‘minutes’) might have occurred due to different handling times of the tissues. Moreover, testing of day 0 samples could only start within 2 hrs after sacrificing the animal, which was caused by the logistical problem of the abattoir and testing unit not being in the same place. Future studies might limit this latency by testing the samples on-site. The brains were submerged into normal saline solution between retrieval and testing. Future studies should clarify if storage in saline alters the biomechanical properties of the tested samples. If so, samples that were stored in saline for up to 4 days might have been predominantly impacted.

## Conclusion

The biomechanical properties of brain tissue can discriminate between day 0 and at least 1-day-old samples with high accuracy using rheometry tests. This supports the potential value of biomechanical analyses for forensic time since death estimations. The receiver operator characteristic analyses between day zero and the pooled data of days one to four show that a PMI of at least 1 day can be determined with a sensitivity of 90%, a specificity of 92%, and a positive likelihood ratio as high as 10.8 using a complex shear modulus cut-off value of 1461 Pa for cerebellar samples. A critical advantage of the here presented method over traditional methods to estimate the time since death is its usability in cases of disintegrated bodies, e.g. when just the head is found.

### Supplementary Information

Below is the link to the electronic supplementary material.

Supplementary file1 (XLSX 12 KB)

## Data Availability

The data is available on request.
